# An ethnobotany of the Lukomir Highlanders of Bosnia & Herzegovina

**DOI:** 10.1186/s13002-015-0068-5

**Published:** 2015-11-25

**Authors:** Jonathan Ferrier, Lana Saciragic, Sabina Trakić, Eric C. H. Chen, Rachelle L. Gendron, Alain Cuerrier, Michael J. Balick, Sulejman Redžić, Emira Alikadić, John T. Arnason

**Affiliations:** Department of Biology, University of Ottawa, Gendron Hall Room 160, 30 Marie Curie, Ottawa, K1N 6 N5 ON Canada; Institute of Economic Botany, The New York Botanical Garden, 200th Street and Kazimiroff Blvd, The Bronx, NY 10458-5126 USA; Department of Chronic Diseases, OHRI The Ottawa Hospital, University of Ottawa OBGYN Suite 8425, 501 Smyth Rd, Ottawa, K1Y 4E9 ON Canada; Emcarta Inc., 32 Lewis Street, Ottawa, K2P 0S3 ON Canada; Department of Obstetrics and Gynecology, University of Ottawa / the Ottawa Hospital, 501 Smyth Road, Ottawa, K1H 8 L6 ON Canada; Department of Botany, Center of Ecology and Natural Resources, University of Sarajevo, Hrasnička cesta 15, 71210 Sarajevo, Bosnia and Herzegovina; Université de Montréal, Institut de recherche en biologie végétale, Jardin botanique de Montréal 4101 rue Sherbrooke Est, Montréal, H1X 2B2 QC Canada; Foundation GEA+, Kranjčevićeva 41, 71000 Sarajevo, Bosnia and Herzegovina

**Keywords:** Postwar ethnobotany, Lukomir highlanders, Bosnia and Herzegovina, Medicinal, food, and endemic plants

## Abstract

**Background:**

This aim of this study is to report upon traditional knowledge and use of wild medicinal plants by the Highlanders of Lukomir, Bjelašnica, Bosnia and Herzegovina (B&H). The Highlanders are an indigenous community of approximately 60 transhumant pastoralist families who speak Bosnian *(Bosanski)* and inhabit a highly biodiverse region of Europe. This paper adds to the growing record of traditional use of wild plants within isolated communities in the Balkans.

**Methods:**

An ethnobotanical study using consensus methodology was conducted in Lukomir in Bjelašnica’s mountains and canyons. Field work involved individual semi-structured interviews during which informants described plants, natural product remedies, and preparation methods on field trips, garden tours, while shepherding, or in settings of their choice. Plant use categories were ranked with informant consensus factor and incorporated into a phylogenetic tree. Plants cited were compared to other ethnobotanical surveys of the country.

**Results:**

Twenty five people were interviewed, resulting in identification of 58 species (including two subspecies) from 35 families, which were cited in 307 medicinal, 40 food, and seven material use reports. Individual plant uses had an average consensus of five and a maximum consensus of 15 out of 25. There were a number of rare and endangered species used as poisons or medicine that are endemic to *Flora Europaea* and found in Lukomir. Ten species (including subspecies) cited in our research have not previously been reported in the systematic ethnobotanical surveys of medicinal plant use in B&H: (*Elymus repens* (L.) Gould, *Euphorbia myrsinites* L., *Jovibarba hirta* (L.) Opiz, *Lilium bosniacum* (Beck) Fritsch, *Matricaria matricarioides* (Less.) Porter ex Britton, *Phyllitis scolopendrium* (L.) Newman, *Rubus saxatilis* L., *Silene uniflora* Roth ssp. *glareosa* (Jord.) Chater & Walters, *Silene uniflora* Roth ssp. prostrata (Gaudin) Chater & Walters, *Smyrnium perfoliatum* L.). New uses not reported in any of the aforementioned systematic surveys were cited for a total of 28 species. Thirteen percent of medicinal plants cited are endemic: *Helleborus odorus* Waldst. et Kit., *Gentiana lutea* L., *Lilium bosniacum* (Beck) Fritsch, *Silene uniflora* Roth ssp. *glareosa* (Jord.) Chater & Walters., *Silene uniflora* Roth ssp. *prostrata* (Gaudin) Chater & Walters, *Salvia officinalis* L., *Jovibarba hirta* (L.) Opiz, and *Satureja montana* L.

**Conclusions:**

These results report on the cohesive tradition of medicinal plant use among healers in Lukomir, Bosnia and Herzegovina. This work facilitates the community’s development by facilitating local and international conversations about their traditional medicine and sharing insight for conservation in one of Europe’s most diverse endemic floristic regions, stewarded by one of Europe’s last traditional Highland peoples.

## Background

The aim of this study is to report upon traditional knowledge and use of wild medicinal plants by the Highlanders of Lukomir, Bjelala, B, Bosnia and Herzegovina (B&H). The project began as a collaboration with the late ethnobotanical authority in B&H, Professor Sulejman Redžić (1954–2013) and was funded by a post war development grant from the Canadian International Development Agency (CIDA). The first ethnobotanical study published from Lukomir was contributed in Redžić’s honour [1]. The project was designed to help describe Lukomir’s biocultural region with the goal of assisting B&H to meet environment and health mandates of the European Union Stabilization and Association Agreement.

**Fig. 1:**
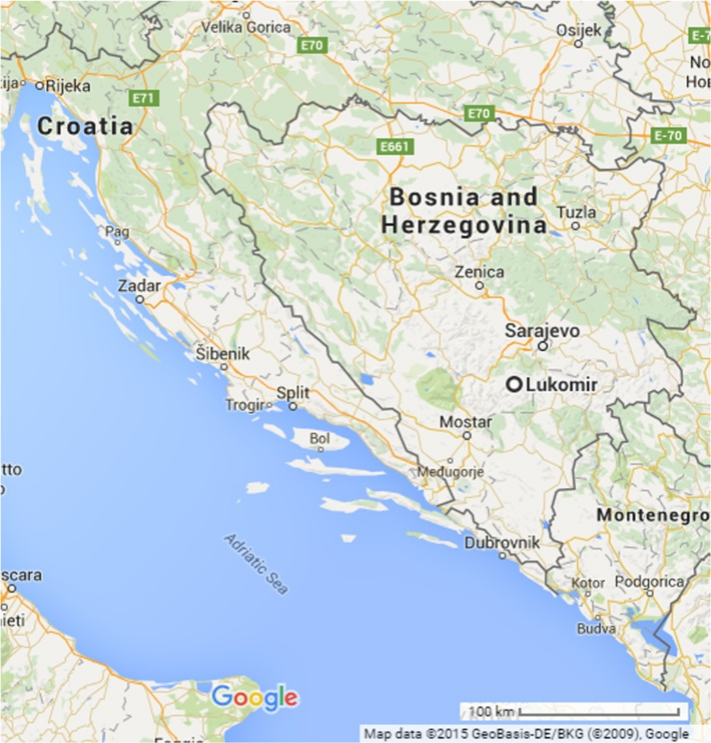
Map of Bosnia and Herzegovina with Lukomir southwest of Sarajevo. Mapdata © 2015 GeoBasis-DE/BKG (© 2009), Google

Lukomir is an isolated village located about 50 km southwest of the capital city, Sarajevo (Fig. 1). Lukomir is situated above Rakitnica canyon with only one access road, which is impassable for part of the year. This isolation has resulted in the preservation of many aspects of traditional lifestyle in Lukomir, including traditional architecture, clothing, herding and medicinal plant gathering and usage [2].

**Fig. 2:**
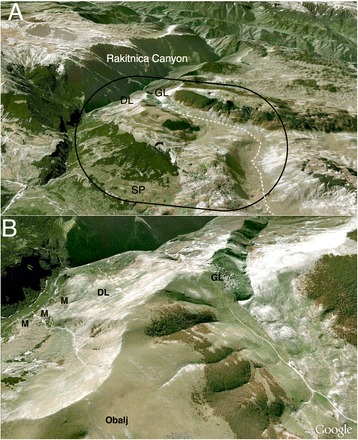
Maps of the Lukomir Highlander ethnobotanical study site. Panel **a** represents the visited study sites and panel **b** is zoomed in to demostrate the *vodenica mlinis*(M) and the Obalj water source's proximity to Donji Lukomir (DL), Gornji Lukomir (GL), and the road (dotted white line)

The Lukomir Highlanders are Bosniak and speak Bosnian (*Bosanski)*. They are one of Europe’s indigenous communities whose members still occasionally practice a traditional transhumant pastoralist lifestyle. They have inhabited the Lukomir territory (Figs. 1 and 2) within the Dinaric Alps for centuries and are historically Bogomil (Sulejman Redžić, personal communication). Autosomal STR loci studies link Lukomir to the isolated Adriatic islands of Brač, Hvar, and Korčula in Croatia [3]. The Highlanders also have a historic connection to transhumant peoples from the Podveležje Plateau near Mostar, B&H, who travelled to Bjelašnica with their livestock in search of water during the summer months [4].

Until recently, shepherds with hundreds in their flocks set out on grazing journeys throughout the Lukomir territory and to neighbouring regions, especially Konjic, B&H. In the wild, shepherds slept with their sheep flocks while other Lukomir family members gathered and cultivated for enough food and fodder to last the long winter.

Since the Bosnian War (1992–5), many inhabitants have moved to cities and herding has declined. Instead, there is a seasonal migration of former inhabitants to Lukomir to gather medicinal plants, make hay during the summer months, and carry on other traditional practices [2]. According to local informants, the number of people staying in Lukomir all winter and the number of people returning in the summer is declining. In the winter of 2007, three families stayed for the winter. In 2008 and 2009, only two families remained all winter. The winter of 2011–2012 was the first winter without inhabitants. This transition in Lukomir’s cultural history was likely exacerbated by 15 m high snowdrifts, an impassable road, and relocations for work and education.

### Healthcare in Lukomir

From 1950 onward, the development of the only road prompted the Highlanders to gradually relocate from Donji Lukomir (Lower Lukomir) to their current location of Gornji Lukomir (Upper Lukomir), which is now generally referred to as Lukomir (see DL and GL in Fig. 2). Before this road**,** Donji Lukomir provided the most efficient access to health and trade services in neighbouring regions via the Rakitnica canyon trails. Lukomir is without a primary healthcare facility, but is known to practice traditional medicine, according to Professor Sulejman Redžić.

### Biodiversity in Lukomir

Approximately 60 % of all vascular plant species listed in the *Flora Europaea* are found in the Balkan Peninsula, making Lukomir part of a highly biodiverse region of Europe [5, 6]. Many other studies of traditional plant use for medicine and nutrition have been conducted in the Balkans, including in B&H. Three key systemic ethnobotanical surveys of B&H have recently identified hundreds of plants and thousands of preparations used in traditional human therapy [6–8]. Many of these plants are used in official pharmacopeias while others are less known and invite further ethnopharmacological studies. Many of the same uses and preparations were found across regions and ethnic groups, showing an established base of medicinal plant use. This study joins a growing collection of research whose goal is to provide an inventory and increase understanding of medicinal plants used in the Balkan Peninsula [9], including countries such as Albania [10–12], Montenegro [13], Kosovo [14], Serbia [15, 16], Bulgaria [17, 18], and Macedonia [19, 20].

## Methods

### Partnership, permits, and prior informed consent

This ethnobotanical study was conducted between 2007 and 2013 with the Lukomir community in Bosnia and Herzegovina, and J.F. and L.S.’s contracting agency. An international collaboration was developed with the Partnerships for Tomorrow Program, Phase II (PTP) funded by the Canadian International Development Agency (CIDA). Ferrier and Šačiragić were hosted by the University of Sarajevo and Foundation GEA+. Research permits were issued by the Municipality of Konjic and the University of Ottawa Human Ethics Review Board (H05-09-07), with prior informed consent from Lukomir’s leaders and informants. The project was compliant with Canada’s tri-council guideline on research with vulnerable populations.

### Study site: Lukomir, Municipality of Konjic, Bosnia & Herzegovina, Europe

An ethnobotanical study using consensus methodology was conducted with the Lukomir Highlanders of B&H in the highlands of Bjelašnica, located southwest of the capital city Sarajevo (Fig. 1). The Lukomir biocultural area is an alpine biogeographic region that is bordered closely by Mediterranean and Continental biogeographic regions within the biocultural area [21]. Many community members are descendants of a Bogomil lineage who first settled in Donji Lukomir (Lower Lukomir) (43.632 lat., 18.194 long., 1200 m a.s.l.) and eventually moved to Gornji Lukomir (Upper Lukomir, commonly referred to as Lukomir) (43.637 lat., 18.182 long., 1460 m a.s.l.) (Fig. 2).

We visited Lukomir repeatedly, in the summers of 2008 (June, July, August), 2009 (August), 2010 (June), and 2012 (August). By volunteering our help to the Highlanders while shepherding, collecting food, and stacking hay, we had more time for interviews and recruited 25 informants from Lukomir who described plants on mountain and canyon field trips, garden tours, while shepherding, or in settings of their choice. The informants were community healers who ranged in age from younger adults to elders. Fifty-six percent were women. Notes and photos were taken when participants chose to display preparation methods of plant and natural remedies. Plant vouchers, the authors’ working field guide, and an iPad (Apple, Cupertino, USA) were used to display collections and photos to elders who could not venture over the mountainside, or for informant review purposes [22].

Land mines were avoided by consulting with a map from the Bosnia and Herzegovina Mine Action Center [23]. Only parts of the Lukomir territory were surveyed by BH MAC and so all plants were collected on trail sides or in areas that were constantly travelled by sheep herds.

Field work followed consensus methodology with individual semi-structured interviews during which L.S., J.F., and S.R. collected the following data: (1) specimen voucher number, (2) photo number, (3) common name, (4) scientific name, (5) family, (6) GPS coordinates, (7) altitude (m a.s.l.), (8) habitat, (9) sub-habitat, (10) flowering time and description, (11) medically active collection time, (12) use, (13) use category, (14) plant part used, (15) amount used, (16) medicine preparation method, (17) medicine administration method, (18) medicine dosing regimen, (19) ethnographic details, (20) informant name and number, and (21) date. Duplicate vouchers were collected (when sustainable) for deposition at the University of Sarajevo Herbarium and the University of Ottawa herbarium, OTT (Ferrier 351–458).

While this work was focused on medical plant use, a non-exhaustive record of food and material uses was made in association with the medicinal plants. Due to the expense and time associated with ethnobotanical projects, we felt that including the food and material mentions would be of greater value to the Lukomir community.

### Ethnobotanical analysis and statistics

#### Human selection of plants

Consensus on an individual usage was the number of informants using the plant out of 25. For categories of phytochemical and pharmacological interest, the informant consensus factor (*F*_*ic*_) function, created by Trotter and Logan [24], was used to supply a numerical ranking factor based on the number of use reports (*n*_*ur*_) and the number of taxa (*n*_*taxa*_) per usage category.

$$ {F}_{ic} = \left({n}_{ur}\mathit{\hbox{-}}\ {n}_{taxa}\right)/\left({n}_{ur}\mathit{\hbox{--}}\  1\right) $$

### Statistics and phylogenetic tree

**Fig. 3:**
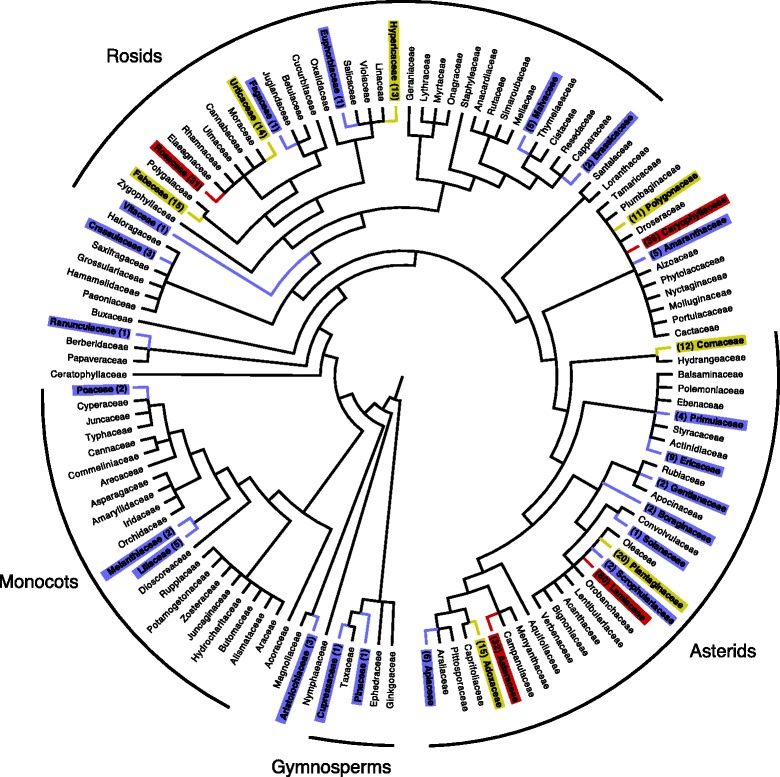
A phylogenetic tree displaying plant families’ use reports within the flora of B&H [25]. Plant families with use reports are in bold. The bracketed numbers beside the families indicate the total number of use reports for that family and are color coded: blue - under 10 reports; yellow - between 10 and 20 reports; red - over 30 reports. Branch lengths are not to scale

Statistical analysis was conducted using Prism 6 software. A phylogenetic tree was constructed using TreeGraph 2.0.47–206 beta, FigTree v1.4.0 (Fig. 3). Taxa are based on the flora of B&H [25] and corrected to match accepted names in Tropicos [26] and the topology presented is based on Angiosperm Phylogeny Group III [27].

## Results and discussion

### Interviews

During our interviews, the Highlanders identified 58 species (including 2 subspecies) from 35 families, which were cited in 307 medicinal, 40 food, and seven material use reports and are included Table 1. Grassland habitats generated the most use reports of plants. Medicinal plant collection was greatest from mid-July to early August as many healers collected plants while in the grasslands harvesting hay.

**Table 1:** Medicinal, food, and material taxa used by the Lukomir Highlanders of Bosnia & Herzegovina

Family	Genus species	†	*	Common name	Habitat	Syntaxa	f	VN
Adoxaceae	*Sambucus nigra* L.			Zova, Zobovina, Elder	Village & shepherd trails	F	11	354
Adoxaceae	*Sambucus wightiana* Wall. ex Wight & Arn*.*			Haptovina	Mountainside slope	Ea, Art	1	376
Adoxaceae	*Sambucus wightiana* Wall. ex Wight & Arn*.*			Haptovina	Mountainside slope	Ea, Art	3	376
Amaranthaceae	*Chenopodium bonus*-*henricus* L.			Šćir, Good King Henry	Village & shepherd trails	Ch, O	5	370
Apiaceae	*Smyrnium perfoliatum* L.			Ljaljica, Perfoliate alexanders	Grassland	O, Arr	2	380
Apiaceae	*Smyrnium perfoliatum* L.			Ljaljica, Perfoliate alexanders	Grassland	O, Arr	2	380
Apiaceae	*Smyrnium perfoliatum* L.			Ljaljica, Perfoliate alexanders	Grassland	O, Arr	2	380
Aristolochiaceae	*Asarum europaeum* L.			Kopitnik/Kopitnjak, European wild ginger	Deciduous forest	F, Qp, O-Co	1	382
Aristolochiaceae	*Asarum europaeum* L.			Kopitnik/Kopitnjak, European wild ginger	Deciduous forest	F, Qp, O-Co	2	382
Aspleniaceae	*Phyllitis scolopendrium* (L.) Newman			Podrebnica (male and female) indicated by sori	Riparian	F, Amph	10	357
Asteraceae	*Achillea millefolium* L.			Kunica, Yarrow	Village & shepherd trails	Arr, Pm, Ch	9	358
Asteraceae	*Achillea millefolium* L.			Kunica, Yarrow	Village & shepherd trails	Arr, Pm, Ch	1	358
Asteraceae	*Artemisia absinthium* L.			Pelin, Absinthe wormwood	Mountainside slope	Art, O	1	396
Asteraceae	*Cichorium intybus* L.			Konjanik, Chicory	Village & shepherd trails	Art, O, Ag	1	411
Asteraceae	*Matricaria matricarioides* (Less.) Porter ex Britton			Kamilica, Stomaklija, Pineappleweed	Village & shepherd trails	Pm	1	351
Asteraceae	*Matricaria matricarioides* (Less.) Porter ex Britton			Kamilica, Stomaklija, Pineappleweed	Village & shepherd trails	Pm	1	397
Asteraceae	*Matricaria matricarioides* (Less.) Porter ex Britton			Kamilica, Stomaklija, Pineappleweed	Village & shepherd trails	Pm	12	351
Asteraceae	*Matricaria matricarioides* (Less.) Porter ex Britton			Kamilica, Stomaklija, Pineappleweed	Village & shepherd trails	Pm	1	351
Asteraceae	*Taraxacum officinale* F.H. Wigg.			Maslačak, Dandelion	Village & shepherd trails	Arr, O, Ag, Pm	1	412
Asteraceae	*Tussilago farfara* L.			Podbijel (♂ or ♀), Coltsfoot	Riparian	O,Pm	4	371
Boraginaceae	*Symphytum officinale* L.			Gavez, Common comfrey, Boneset	Riparian	Ag, Bid, Art	2	383
Brassicaceae	*Capsella bursa-pastoris* (L.) Medik.			Rustemača, Shepherd’s purse	Village & shepherd trails	Ch, O, Art	2	398
Caryophyllaceae	*Silene uniflora* Roth ssp. *glareosa* (Jord.) Chater & Walters	†		Puca, Sea campion	Grassland	Arr, Be, O-Co	2	353
Caryophyllaceae	*Silene uniflora* Roth ssp. *glareosa* (Jord.) Chater & Walters	†		Puca, Sea campion	Grassland	Arr, Be, O-Co	7	353
Caryophyllaceae	*Silene uniflora* Roth ssp. *glareosa* (Jord.) Chater & Walters	†		Puca, Sea campion	Grassland	Arr, Be, O-Co	1	353
Caryophyllaceae	*Silene uniflora* Roth ssp. *glareosa* (Jord.) Chater & Walters	†		Puca, Sea campion	Grassland	Arr, Be, O-Co	8	353
Caryophyllaceae	*Silene uniflora* Roth ssp. *prostrata* (Gaudin) Chater & Walters	†		Puca, Sea campion	Grassland	Arr, Be, O-Co	8	350
Caryophyllaceae	*Silene uniflora* Roth ssp. *prostrata* (Gaudin) Chater & Walters	†		Puca, Sea campion	Grassland	Arr, Be, O-Co	2	350
Caryophyllaceae	*Silene uniflora* Roth ssp. *prostrata* (Gaudin) Chater & Walters	†		Puca, Sea campion	Grassland	Arr, Be, O-Co	7	350
Caryophyllaceae	*Silene uniflora* Roth ssp. *prostrata* (Gaudin) Chater & Walters	†		Puca, Sea campion	Grassland	Arr, Be, O-Co	1	350
Cornaceae	*Cornus mas* L.			Drijen, European dogwood	Deciduous forest	O-Co, F,Qp,Ps	2	384
Cornaceae	*Cornus mas* L.			Drijen, European dogwood	Deciduous forest	O-Co, F,Qp,Ps	2	384
Cornaceae	*Cornus mas* L.			Drijen, European dogwood	Deciduous forest	O-Co, F,Qp,Ps	2	384
Cornaceae	*Cornus mas* L.			Drijen, European dogwood	Deciduous forest	O-Co, F,Qp,Ps	2	384
Cornaceae	*Cornus mas* L.			Drijen, European dogwood	Deciduous forest	O-Co, F,Qp,Ps	2	384
Cornaceae	*Cornus mas* L.			Drijen, European dogwood	Deciduous forest	O-Co, F,Qp,Ps	2	384
Crassulaceae	*Jovibarba hirta* (L.) Opiz	†		Čuvarkuća, Beard of Jupiter, Hen and Eggs	Rockland	Amph	1	379
Crassulaceae	*Jovibarba hirta* (L.) Opiz	†		Čuvarkuća, Beard of Jupiter, Hen and Eggs	Rockland	Amph	1	379
Crassulaceae	*Sedum sexangulare* L.			Zednjak, Tasteless stonecrop	Rockland	Amph	1	416
Cupressaceae	*Juniperus communis* L.			Smreka, Common juniper	Mountainside slope	Jun, O-Co	1	399
Equisetaceae	*Equisetum arvense* L.			Preslica, Field horsetail	Riparian	Pm, Ch	3	367
Equisetaceae	*Equisetum arvense* L.			Preslica, Field horsetail	Riparian	Pm, Ch	2	367
Ericaceae	*Vaccinium myrtillus* L.			Borovnica, European blueberry	Mountainside slope	V, V-P	1	368
Ericaceae	*Vaccinium myrtillus* L.			Borovnica, European blueberry	Mountainside slope	V, V-P	5	368
Ericaceae	*Vaccinium vitis-idaea* L.			Brusnica, Lingonberry	Mountainside slope	V	1	385
Ericaceae	*Vaccinium vitis-idaea* L.			Brusnica, Lingonberry	Mountainside slope	V	2	385
Euphorbiaceae	*Euphorbia myrsinites* L.			Mliječavac, Myrtle spurge	Rockland	Af, S-Ch, Be	1	400
Fabaceae	*Anthyllis vulneraria* L.			Ranjenik, Kidney vetch	Grassland	Be, St	4	372
Fabaceae	*Anthyllis vulneraria* L.			Ranjenik, Kidney vetch	Grassland	Be, St	4	372
Fabaceae	*Ononis spinosa* L.			Gladišika, Spiny restharrow	Grassland	Be, S-Ch, Ps	3	377
Fabaceae	*Trifolium pratense* L.			Crvena djetelina, Red clover	Grassland	Arr, Ag, Be	2	386
Fabaceae	*Trifolium repens* L.			Bijela djetelina, White clover	Grassland	Ag, M, Arr	2	387
Fagaceae	*Fagus sylvatica* L.			Bukva, European beech	Deciduous forest	F	1	410
Gentianaceae	*Gentiana lutea* L.	†		Lincura, Great yellow gentian	Grassland	St, Pim	1	393
Gentianaceae	*Gentiana lutea* L.	†		Lincura, Great yellow gentian	Grassland	St, Pim	1	393
Hypericaceae	*Hypericum perforatum* L.			Kantarion, Ženska trava, St. John’s wort	Grassland	Orig, Ps, Be	2	355
Hypericaceae	*Hypericum perforatum* L.			Kantarion, Ženska trava, St. John’s wort	Grassland	Orig, Ps, Be	11	355
Lamiaceae	*Mentha longifolia* (L.) L.			Metvica, Nana, Horse mint	Riparian	Bid, Mol, M-C	1	413
Lamiaceae	*Mentha longifolia* (L.) L.			Metvica, Nana, Horse mint	Riparian	Bid, Mol, M-C	1	401
Lamiaceae	*Mentha longifolia* (L.) L.			Metvica, Nana, Horse mint	Riparian	Bid, Mol, M-C	15	349
Lamiaceae	*Mentha pulegium* L.			Verem, European pennyroyal	Riparian	Bid, Mol, M-C	1	402
Lamiaceae	*Nepeta cataria* L.			Macina trava, Catnip	Rockland	O, Be	5	369
Lamiaceae	*Nepeta cataria* L.			Macina trava, Catnip	Rockland	O, Be	5	369
Lamiaceae	*Nepeta cataria* L.			Macina trava, Catnip	Rockland	O, Be	5	369
Lamiaceae	*Salvia officinalis* L.	†		Kadulja, Sage	Mountainside slope	S-Ch, O-Co	15	348
Lamiaceae	*Salvia officinalis L.*	†		Kadulja, Sage	Mountainside slope	S-Ch, O-Co	1	348
Lamiaceae	*Satureja montana* L.			Vrijesak, Winter savory	Rockland	S-Ch, O-Co	6	366
Lamiaceae	*Satureja montana* L.	†		Vrijesak, Winter savory	Rockland	S-Ch, O-Co	1	366
Lamiaceae	*Teucrium montanum* L.			Iva, Mountain germander	Rockland	Be, S-Ch	12	352
Lamiaceae	*Thymus serpyllum* L.			Čubra (Crvena i Bijela), Majčina dušica, Breckland thyme	Rockland	Be, S-Ch	5	365
Lamiaceae	*Thymus serpyllum* L.			Čubra (Crvena i Bijela), Majčina dušica, Breckland thyme	Rockland	Be, S-Ch	7	365
Liliaceae	*Convallaria majalis* L.			Dudurika, Lily of the vally	Deciduous forest	Qp, F, O-Co	3	378
Liliaceae	*Lilium bosniacum* (Beck) Fritsch	†		Bosanski ljiljan, Zlatan, Bosnian lily	Grassland	Arr, Be, O-Co	1	417
Liliaceae	*Lilium bosniacum* (Beck) Fritsch	†		Bosanski ljiljan, Zlatan, Bosnian lily	Grassland	Arr, Be, O-Co	1	417
Malvaceae	*Tilia platyphyllos* Scop.			Lipa, Large-leaved linden	Grassland	F, Qp	4	374
Malvaceae	*Tilia platyphyllos* Scop.			Lipa, Large-leaved linden	Grassland	F, Qp	1	374
Melanthiaceae	*Veratrum album* L.			Čemerika, European white hellebore	Grassland	Ad, Arr	2	388
Parmeliaceae	*Cetraria islandica* (L.) Ach			Islandski lišaj, Mašina, Iceland moss	Mountainside slope	St, Be	1	403
Pinaceae	*Pinus nigra* Arnold			Bor, Black pine	Mountainside slope	St, Be	1	418
Plantaginaceae	*Plantago lanceolata* L.			Bokvica ♀, Ribwort plantain	Grassland	Arr, Ag, Be	1	359
Plantaginaceae	*Plantago lanceolata* L.			Bokvica ♀, Ribwort plantain	Grassland	Arr, Ag, Be	9	359
Plantaginaceae	*Plantago major* L.			Bokvica, Broadleaf plantain	Village & shepherd trails	Pm, Ch	1	360
Plantaginaceae	*Plantago major* L.			Bokvica, Broadleaf plantain	Village & shepherd trails	Pm, Ch	9	360
Poaceae	*Elymus repens* (L.) Gould			Pirika, Couch grass	Grassland	Pm, Ag, Arr	1	389
Poaceae	*Elymus repens* (L.) Gould			Pirika, Couch grass	Grassland	Pm, Ag, Arr	1	389
Polygonaceae	*Polygonum bistorta* L.			Srčanik, Bistort	Grassland	Ad, Arr	11	356
Primulaceae	*Primula veris* L.			Jagorčevina, Cowslip	Grassland	Arr, Ad, Ps	4	373
Ranunculaceae	*Helleborus odorus* Waldst. & Kit. ex Willd.	†		Kukurijek, Hellebore	Grassland	Ps, Cor, F, Qp	1	394
Rosaceae	*Crataegus monogyna* Jacq.			Glog, Gloginje, Hawthorn	Deciduous forest	Ps, Cor, Qp	2	361
Rosaceae	*Crataegus monogyna* Jacq.			Glog, Gloginje, Hawthorn	Deciduous forest	Ps, Cor, Qp	8	361
Rosaceae	*Prunus spinosa* L.			Trn, Trnjina, Blackthorne Rakija, Brandy	Mountainside slope	Ps, F, Qp	1	405
Rosaceae	*Prunus spinosa* L.			Trn, Trnjina, Blackthorne Rakija, Brandy	Mountainside slope	Ps, F, Qp	1	405
Rosaceae	*Prunus spinosa* L.			Trn, Trnjina, Blackthorne Rakija, Brandy	Mountainside slope	Ps, F, Qp	1	405
Rosaceae	*Rosa glauca* Pourr.			Šipak/Šipina	Mountainside slope	O-Co, Orig	8	364
Rosaceae	*Rosa glauca* Pourr.			Šipak/Šipina, Redleaf rose	Mountainside slope	O-Co, Orig	8	364
Rosaceae	*Rubus idaeus* L.			Malina, Raspberry	Deciduous forest	Ea, Atr	1	406
Rosaceae	*Rubus saxatilis* L.			Kupina, Stone bramble	Grassland	Af, S-Ch	1	407
Scrophulariaceae	*Verbascum thapsus* L.			Divizbina, Divizma, Great mullein	Grassland	Art, O	2	390
Solanaceae	*Solanum tuberosum* L.		*	Krompir, Potato	Cultivated	Cult, Ch	1	408
Urticaceae	*Urtica dioica* L.			Žara, Kopriva, Stinging nettle	Village & shepherd trails	Art, O	1	362
Urticaceae	*Urtica dioica* L.			Žara, Kopriva, Stinging nettle	Village & shepherd trails	Art, O	1	362
Urticaceae	*Urtica dioica* L.			Žara, Kopriva, Stinging nettle	Village & shepherd trails	Art, O	5	362
Urticaceae	*Urtica dioica* L.			Žara, Kopriva, Stinging nettle	Village & shepherd trails	Art, O	7	362
Vitaceae	*Vitis vinifera* L.		*	Sirće, Vinegar	Cultivated	Cult	1	409

Table 1 details the classification, names, habitat, frequency of use reports and voucher numbers of the 58 species (including 2 subspecies) identified by the Highlanders during this study*Ad* Adiantetalia, *Af* Arabidetalia flavescentis, *Ag* Agrostiealia, *Amph* Amphoricarpetalia, *Arr* Arrhenatheretalia, *Art* Artemisietalia, *Atr* Atropetalia, *Be* Brometalia erecti, *Bid* Bidentetalia, *Ch* Chenopodietalia, *Cor* Corynephoretalia, *Cult* cultivated, *Ea* Epilobietalia angustifoliae, *F* Fagetalia, *Jun* Juniperetalia, *M-C* Montio-Cardaminetalia, *M* Molinietalia, *Mol* Molinietalia, *O* Onopordetalia, *O-Co* Ostryo-Carpinetalia orientalis, *Orig* Origanetalia, *Pm* Plantaginetalia majoris, *Ps* Prunetalia spinosae, *Qp* Quercetalia pubescentis, *S-Ch* Scorzonero-Chrysopogonetalia, *St* Stipetalia, *V* Vaccinietalia, *V-P* Vaccinio-Piceetalia

A dagger (†) indicates European endemic taxa. An asterisk (*) indicates plants found in a garden rather than the wild

The average consensus on use of medicinal plants was 3.5 informants out of 25, and a maximum consensus of 15 was obtained on the medicinal plants *Mentha longifolia* (L.) L. and *Salvia officinalis* L. Average food plant consensus was three. The maximum food plant consensus was eight for *Urtica dioica* L. Finally, there was a maximum consensus of two for material plants *Pinus nigra* Arnold and *Cornus mas* L.

For a community of approximately 60 households, almost half of the households were interviewed. We wondered if the list of medicinal plants was small; however, when looking at our cross-cultural comparison of Cree, Highlanders and Maya we noted that while Lukomir had fewer use reports than the Maya, they had much more than the Cree [28]. This made sense to us that the Cree had less than Lukomir because of their northern location, less biodiversity and cultural assimilation via residential schools in Canada, and that Lukomir healers had fewer reports than the Maya because the Maya live in a tropical rain forest with much greater biodiversity. Lukomir’s data, within a continental, alpine and Mediterranean geography in the middle of these two extremes felt reasonable to us.

### Frequency of use reports per usage category

**Fig. 4:**
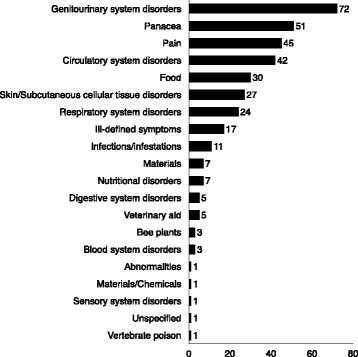
Distribution of medicinal, food, and material plants according to frequency of use reports

The number of use reports per usage category (Fig. 4) varied from 1 to 72. High frequency categories included: genitourinary system disorders, panacea, pain, and circulatory system disorders. Medium frequency categories included: food, skin/subcutaneous cellular tissue disorders, respiratory system disorders, and ill-defined symptoms with many low frequency results (Fig. 4).

The high use of plants for genitourinary system disorders reported (20 %), especially gynaecological uses, may be linked to researcher LŠ and the majority of interviewees being female (*n* = 14 of 25, 56 %), as women would be more likely to have knowledge or experience of these uses and may also have been more comfortable sharing this information with a woman interviewer. However, high female input was predicted based on our survey of the literature [29].

Panacea treatments were highly cited (14 %) since they were often cross-listed as food plants, which included a number of economically important plants. High requests for panacea treatments is probably linked to diabetes, which the visiting physician cited as a top health concern. Diabetic insults affect many tissues and organs, leading to symptoms like frequent urination, neuropathy, and slow healing sores, and panacea treatments are an important resource for Highlanders with these symptoms.

Pain treatment was a high frequency usage (13 %), likely due to hard labor in wet and cold conditions. This may also be linked to heart disease which was another top health concern described by the visiting physician. These high frequency categories indicate that Highlander community members are requesting traditional medicines in line with major health concerns.

### Frequency of taxa per usage category

**Fig. 5:**
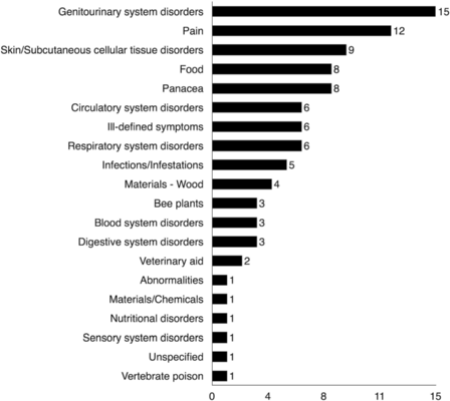
Distribution of medicinal, food, and material plants according to frequency of taxa

Frequency of taxa per usage category distributions revealed the type of usages of most concern (Fig. 5). The medicinal categories employing the largest number of taxa included genitourinary system disorders, pain, and skin/subcutaneous cellular tissue disorders, indicating that these categories are of high community concern. Medium categories included food, panacea, circulatory system disorders, ill-defined symptoms, respiratory system disorders, and infections/infestations and may mark chronic or recurring health conditions that present more often than low frequency categories (four species or less). Table 2 separates the number of taxa into specific uses in their associated usage category.

**Table 2:** The number of taxa for specific uses per usage category

Usage category	Number of taxa
Ailment prophylaxis	1
Bee plants	
Honey	3
Blood system disorders	
Blood clots	1
Circulation	2
Circulatory system disorders	
Heart trouble	5
Digestive system disorders	
Diarrhoea	1
Hard stomach in children	1
Liver aid	1
Food	
Vegetables	4
Beverage	1
Candy	1
Preserve, jam	2
Coffee substitute	1
Genitourinary system disorders	
Female infertility	1
Genitourinary system health	5
Kidney and bladder pain/stones	2
Urinary tract and kidney health	4
Heavy or irregular periods	2
Infection	3
Urinary tract and kidney infection/cleanser	1
Vaginal discharge	2
Ill-defined symptoms	
Internal medicine	6
Infections/infestations	
Cold symptoms	1
Wounds	1
Fever	3
Materials	
Flute and violin	1
Furniture	1
Rake	1
Shingle	1
Tools	1
Materials/Chemicals	
Teeth cleaning	1
Nutritional disorders	
Nutritional deficiency/low iron	1
Pain	
Chest pain	2
Ear pain (children)	1
Eye pain	1
Headache	2
Menstrual cramps and stomach pain	1
Pain under ribs	1
Stomach pain	5
Panacea	
Cure all	8
Respiratory system disorders	
Asthma cough and chest pains	3
Cough/infection	4
Skin/Subcutaneous cellular tissue disorders	
Condylomata acuminata (warts)	2
Skin infection and irritations	1
Cuts and infections	5
Swollen skin and fat deposits under the skin	1
Unspecified	
Unspecified	1
Vertebrate poison	
Chicken poison	1
Veterinary aid	
Antivenin for sheep	1
Nit shampoo for sheep	1

Table 2 presents the number of taxa assigned specific uses and their associated usage category

High diversity (five or more taxa) was found in the following uses: panacea, internal medicine, heart trouble, genitourinary system health, stomach pain, cuts, and infections.

### Informant consensus factor (*F*_*ic*_) per usage category

**Fig. 6:**
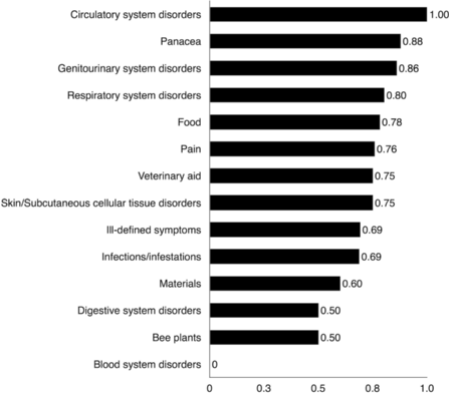
Distribution of medicinal, food, and material plants according to frequency of *F*_*ic*_ values

For a disease level analysis, the *F*_*ic*_ values were calculated (Fig. 6). Here, 10 of the 15 categories had a *F*_*ic*_ value ≥0.69, indicating a high degree of consensus among Lukomir’s healers. The average *F*_*ic*_ was 0.64; to compare human medicinal plant use cross-culturally, non-medicinal categories (bee plants, materials, veterinary aids, food) were removed from this Lukomir survey for an average *F*_*ic*_ of 0.76.

These values are high in comparison to a study in East Timor where the average *F*_*ic*_ values calculated for medicinal plant use categories were 0.30 (Laklei culture) and 0.08 (Idate culture) [30]. The very low value for Idate culture may be due to their culture of secrecy surrounding medicinal plant use. Both Lukomir averages were also greater than averages calculated from a study by Amiguet et al. [31], with the Q’eqchi’ Maya of Belize (average *F*_*ic*_ = 0.62), and the study with the Yucatec Maya of Mexico (*F*_*ic*_ = 0.48) [32]. This difference in *F*_*ic*_ results may be explained by recognizing that the Maya informants were from isolated villages while the Lukomir Highlanders, although transhumant, share a single village where they traditionally stayed all winter, snow-locked, and where the interviews were conducted.

These results indicate that these plants are well known in a well defined community-based traditional knowledge system. When consensus is high, these categories are more likely to be active in condition-related pharmacological assays [33].

### Analysis of medicinal plant families

**Fig. 7:**
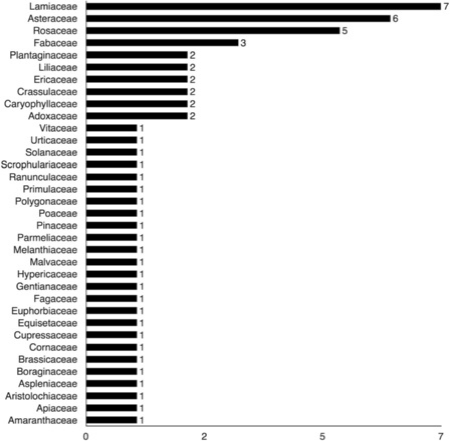
Frequency of species per family

As a first step to identifying key plant families, the number of medicinal, food, and material species by family was ranked (Fig. 7). The three most diverse families were the Lamiaceae (7 species), Asteraceae (6), and Rosaceae (5).

Four medicinal taxa were cross listed as foods, showing that medicinal and food plant categories are not considered mutually exclusive. These traditional foods are often cited for having disease preventing properties and the loss of traditional diets is associated with the rise of chronic conditions such as heart disease and diabetes [1, 34].

The phylogeny (Fig. 3) links plant families to their use reports. Among the top three families, Lamiaceae had 80 use reports (77 medicinal) (Table 1). The Lamiaceae in Lukomir flourish in the Mediterranean environment on the south-facing dolomite slopes of the Raktinica Canyon. The Lukomir cultural area is a refuge of wild endemic Lamiaceae taxa, as indicated by our ongoing floristic survey. The Caryophyllaceae family also had high use (36 use reports, all medicinal) (Table 1) and is often found fringing village trails and grasslands. Asteraceae received 32 (30 medicinal) use reports (Table 1), which is not surprising given the family’s cosmopolitan distribution throughout Lukomir’s alpine, Mediterranean, and continental biogeographical regions.

### Traditional medicinal plant preparation

**Fig. 8:**
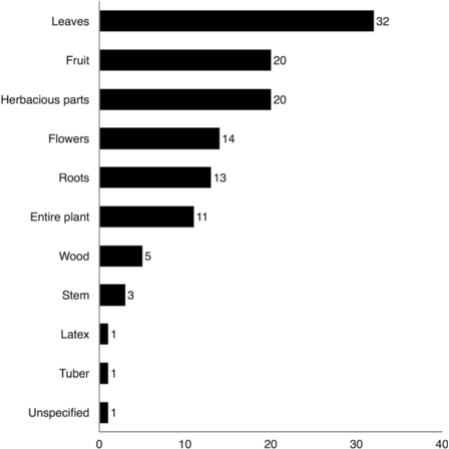
Organ use of the plant species listed in Table 1

**Fig. 9:**
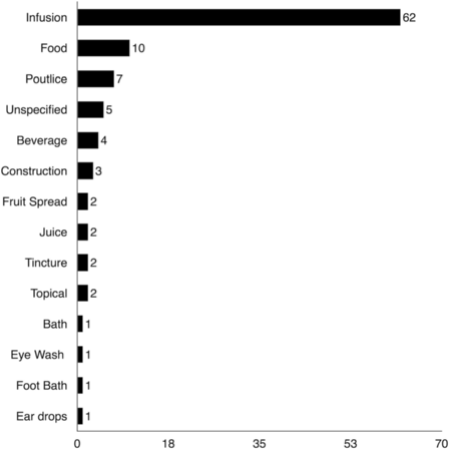
Plant preparation reports of the plant species listed in Table 1

Leaves are the plant part cited most often by the Highlanders (Fig. 8). Infusions were the most common preparation (Fig. 9), as was found in several other studies of B&H [6–8]). Some taxa were included in family *čaj* (infusion) collections, not necessarily viewed as medicine but consumed after mealtime for their general health benefits [1].

### Plant usage compared to other studies in Bosnia & Herzegovina

In comparison with other ethnobotanical surveys of medicinal plants used in B&H [6–8]) ten species (including subspecies) in our research have not previously been reported (other than in our previous publication from the same project [1]): *Elymus repens* (L.) Gould, *Euphorbia myrsinites* L., *Jovibarba hirta* (L.) Opiz, *Lilium bosniacum* (Beck) Fritsch, *Matricaria matricarioides* (Less.) Porter ex Britton, *Phyllitis scolopendrium* (L.) Newman, *Rubus saxatilis* L., *Silene uniflora* Roth ssp. glareosa (Jord.) Chater & Walters, *Silene uniflora* Roth ssp. prostrata (Gaudin) Chater & Walters, *Smyrnium perfoliatum* L. (Table 3). New medicinal uses not reported in any of the aforementioned systematic surveys were cited for a total of 29 species, including the ten newly reported species.

**Table 3:** A comparison of this study’s reported plants with other ethnobotanical studies in Bosnia and Herzegovina

Genus species	Plant usage reported in other studies
*Achillea millefolium* L.	Y [8, 29, 39]
*Anthyllis vulneraria* L.	Y [8, 29, 39]
*Artemisia absinthium* L.	Y [6–8, 29]
*Asarum europaeum* L.	Y [8]
*Capsella bursa-pastoris* (L.) Medik.	Y [6, 8, 29, 39]
*Cetraria islandica* (L.) Ach	Y [6–8]
*Chenopodium bonus*-*henricus* L.	Y [39]
*Cichorium intybus* L.	Y [6, 8, 29, 39]
*Convallaria majalis* L.	Y [8]
*Cornus mas* L.	Y [6, 8, 39]
*Crataegus monogyna* Jacq.	Y [6–8, 29, 39]
*Elymus repens* (L.) Gould	N
*Equisetum arvense* L.	Y [8, 29]
*Euphorbia myrsinites* L.	N
*Fagus sylvatica* L.	Y [8, 39]
*Gentiana lutea* L.	Y [6, 8]
*Helleborus odorus* Waldst. & Kit. ex Willd.	Y [8]
*Hypericum perforatum* L.	Y [6–8, 29, 39]
*Jovibarba hirta* (L.) Opiz	N
*Juniperus communis* L.	Y [6–8]
*Lilium bosniacum* (Beck) Fritsch	N
*Matricaria matricarioides* (Less.) Porter ex Britton	N
*Mentha longifolia* (L.) L.	Y [6–8, 39]
*Mentha pulegium* L.	Y [6–8, 29, 39]
*Nepeta cataria* L.	Y [6, 8, 29]
*Ononis spinosa* L.	Y [6–8, 39]
*Phyllitis scolopendrium* (L.) Newman	N
*Plantago lanceolata* L.	Y [6–8, 39]
*Plantago major* L.	Y [6–8, 39]
*Polygonum bistorta* L.	Y [7, 8, 39]
*Primula veris* L.	Y [8, 39]
*Prunus spinosa* L.	Y [7, 8, 39]
*Rosa glauca* Pourr.	Y [39]
*Rubus idaeus* L.	Y [6–8, 39]
*Rubus saxatilis* L.	N
*Salvia officinalis* L.	Y [6–8, 29, 39]
*Sambucus ebulus* L.	Y [6, 7, 29]
*Sambucus nigra* L.	Y [6–8, 39]
*Satureja montana* L.	Y [6–8, 29, 39]
*Sedum sexangulare* L.	Y [29]
*Silene uniflora* Roth ssp. *glareosa* (Jord.) Chater & Walters	N
*Silene uniflora* Roth ssp. *prostrata* (Gaudin) Chater & Walters	N
*Smyrnium perfoliatum* L.	N
*Solanum tuberosum* L.	Y [6, 7]
*Symphytum officinale* L.	Y [6–8, 29, 39]
*Taraxacum officinale* F.H. Wigg.	Y [6–8, 29, 39]
*Teucrium montanum* L.	Y [6–8, 29]
*Thymus serpyllum* L.	Y [8, 29, 39]
*Tilia platyphyllos* Scop.	Y [6–8]
*Trifolium pratense* L.	Y [6, 7, 39]
*Trifolium repens* L.	Y [6, 7, 39]
*Tussilago farfara* L.	Y [6–8, 29, 39]
*Urtica dioica* L.	Y [6–8, 29, 39]
*Vaccinium myrtillus* L.	Y [6–8, 39]
*Vaccinium vitis-idaea* L.	Y [6, 8, 39]
*Veratrum album* L.	Y [8]
*Verbascum thapsus* L.	Y [7, 8]
*Vitis vinifera* L.	Y [6, 7]

Table 3 presents which plants have been cited in other studies in B&H

### Use of rare and endemic plants

This study considers endemics in the context of Flora Europaea and did not consider garden plants as endemics. Garden plants listed in our survey, such as potato, have been denoted in Table 1. It was observed that Lukomir is a wild refuge for many european endemics. For example, sage was never found in the garden, and was only located once, halfway down Europe’s largest canyon.

The Highlanders use eight endemic plant species: *Helleborus odorus* Waldst. et Kit., *Gentiana lutea* L., *Lilium bosniacum* (Beck) Fritsch, *Silene uniflora* Roth ssp. *glareosa* (Jord.) Chater & Walters., *Silene uniflora* Roth ssp. *prostrata* (Gaudin) Chater & Walters, *Salvia officinalis* L., *Jovibarba hirta* (L.) Opiz, and *Satureja montana* L. Of the total medicinal plant use, 13 % is endemic flora. This is not surprising, since the Lukomir Highlanders are an indigenous community inhabiting one of Europe’s most diverse biological areas [5, 6].

*Lilium bosniacum*, *Gentiana lutea* L., *Silene uniflora* Roth ssp. *prostrata* (Gaudin) Chater & Walters and *Jovibarba hirta* (L.) Opiz, are among the species which have not been listed in previous traditional medicine research in B&H (Table 1).

The Highlanders uses the root of *Gentiana lutea* L. have not been reported in other studies in the country (Table 1).

*Lilium bosniacum* (Beck) Fritsch, the Bosnian Lily, is endemic to the central Dinaric Alps whose taxonomic status has been much debated [35]. Studies have come to differing conclusions as to whether *L. bosniacum* is a distinct species, a subspecies or a variety of *L. carniolicum* [35–37]). Recent molecular cytogenic studies have found evidence to support *L. bosniacum’s* status as a distinct species [35, 37]. According to Tropicos, *Lilium bosniacum* (Beck) Fritsch has species rank [38].

*Lilium bosniacum* has been classified as a rare and vulnerable taxon in the pending edition of the Red Book of B&H. Despite not being its typical habitat, *L. bosniacum* is found in Bjelašnica [35]. One informant in Lukomir cited two uses for *L. bosniacum*.

*Salvia officinalis* L. (Kadulja, sage) was not found in gardens, but growing wild. In nearby Albania, this plant is an important export product to Western Europe and the United States [39]. This plant was cited a large number of times in the 2007 systemic study of the country’s plant use [8].

### Post Bosnian War development, conservation, and the dietary shift in Lukomir

**Fig. 10:**
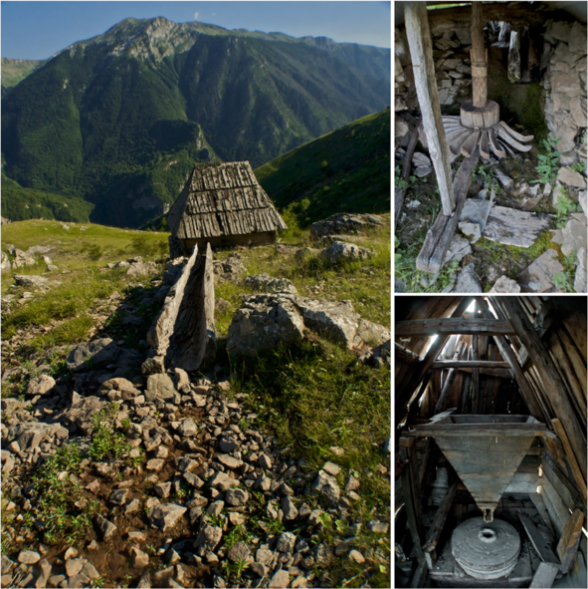
A *vodenica mlini* (hydro mill) on the dried up Vodenica waterway in July

Recent changes have deteriorated the health and environment status in Lukomir. Before 1997, three *vodenica mlini* (hydro powered cereal mills) conducted the process of removing hard seed coats from Lukomir’s cereal crops. The Vodenica waterway runs from the peak of Obalj mountain and down through the canyons of the Rakitnica (Fig. 2). Troughs handcrafted from the hardwood *Fagus sylvatica* L. (*Bukva*, Beech) funneled the *vodenica* and hydro powered the wooden turbines constructed of *F. sylvatica* vanes and axils (Fig. 10). The turbine turned one of the two hand fashioned grinding stones that produced wholegrain cereal flour. Our informants indicated that the cereals routinely grown and milled in Lukomir were: *Avena sativa* L. (*Zob*, Oat), *Secale sereale* L. (*Raž*, Rye), *Hordeum vulgare* L. (*Ječam*, Barley), *Triticum aestivum* L. (*Pšenica*, Wheat), and *Zea mays* L. (*Kukuruz,* Corn).

Once powered by the spring rains and snowmelt, the mills are now in disuse on the edge of Donji Lukomir (see M, Fig. 2). The local cereal crops are no longer cultivated and milled and have been replaced by imported soft white wheat flour. When asked why the *mlini* are not used, informants explained that in 1997, 1 year after the Bosnian War, water diversion began in Lukomir. Water pipelines were placed at the mouth of the Obalj mountain spring to share water with lower elevation communities in the Municipality of Konjic. Following diversion, the waterways began to dry up by mid-June, leaving the mills devoid of running water. There was no hydro power available for milling the June, July, and September cereal harvests. This water work development caused the collapse of the *vodenica mlini,* cultivation of indigenous cereal landraces, and Lukomir’s organic multigrain diet. As running the mills involved considerable physical effort, a source of exercise was also lost.

Water work diversion lead to a community-wide dietary shift. Many locals now depend on a food truck that delivers white flour once per week. The time spent working to produce their own multigrain flour is gone, and the diet and lifestyle shift is contributing to Lukomir’s greatest health concerns: heart disease and diabetes [1].

All of the rare and endemic flora and many other species listed in (Table 1) are found in the area from Obajl (1874 m a.s.l.) to the Rakitnica River (640 m a.s.l.). These areas are now more endangered because of the water work diversion and ongoing habitat loss. Although all people from B&H should have equal and shared access to water, this particular development was placed in the centre of one of the most biogeographically sensitive areas of both the country and continent without sufficient consultation and foresight.

The collapse of Lukomir’s traditional food system comes at a time when tourism is increasing in the community and B&H is modelling itself as an international destination to provide an experience of European traditional culture. The region’s flora and organic multigrain products are popular attractions among hiking enthusiasts and health conscious visitors to the Dinaric Alps. Many women in the community depend on tourism as they open their homes to tourists, serve food, teas, and coffee, and sell their knitted merchandise. Planning for a self-sufficient future for the Lukomir Highlanders should include preservation of the *vodenica mlini* as a unique cultural technology, an attraction for visitors to the area and a contributor to a healthier lifestyle.

## Conclusions

Although post war development has contributed to the erosion of the self-sustaining traditional lifestyle of the Lukomir Highlanders, our results demonstrate that they continue to have a strong traditional medicine and gathered food system. This traditional knowledge must continue to be valued and maintained in planning for a durable, self-sufficient future for the Lukomir Highlanders. In addition, special emphasis should be placed on the preservation of the *vodenica mlini* (hydro cereal mills) - a unique cultural technology and visitor attraction that contributes to a traditionally healthy diet and lifestyle.

## Abbreviations

B&H: Bosnia and Herzegovina
DL: Donji Lukomir
GL: Gornji Lukomir
CIDA: Canadian International Development Agency
BH MAC: Bosnia and Herzegovina Mine Action Centre
F_ic_: Informant consensus factor
